# Ethanolic Extract of *Ganoderma mexicanum* Pat. Mycelium: A Source of Bioactive Compounds with Antiproliferative Activity and Potential PPAR-γ Natural Ligands

**DOI:** 10.3390/ph18060909

**Published:** 2025-06-18

**Authors:** Lucia T. Angulo-Sanchez, Max Vidal-Gutiérrez, Heriberto Torres-Moreno, Martín Esqueda, Aldo Gutiérrez, Georgina Vargas, Juan Luis Monribot-Villanueva, José A. Guerrero-Analco, César Muñoz-Bacasehua, Ramón Enrique Robles-Zepeda

**Affiliations:** 1 Centro de Investigación en Alimentación y Desarrollo, A.C. Carretera Gustavo Enrique Astiazarán Rosas 46, La Victoria, Hermosillo 83304, Sonora, Mexico; lteresa.angulo@gmail.com (L.T.A.-S.); asaldana@ciad.mx (A.G.); gvargas@ciad.mx (G.V.); 2 Departamento de Ciencias Químico, Biológicas y Agropecuarias, Campus Navojoa, Universidad de Sonora, Lázaro Cárdenas del Río 100, Francisco Villa, Navojoa 85880, Sonora, Mexico; max.vidal@unison.mx (M.V.-G.); cesar.munoz@unison.mx (C.M.-B.); 3 Departamento de Ciencias Químico, Biológicas y Agropecuarias, Campus Caborca, Universidad de Sonora, Avenida K SN, Eleazar Ortiz, H. Caborca 83600, Sonora, Mexico; heriberto.torres@unison.mx; 4 Instituto de Ecología A.C. Red de Estudios Moleculares Avanzados, Carretera Antigua a Coatepec 351, Col. El Haya, Xalapa 91070, Veracruz, Mexico; juan.monribot@inecol.mx (J.L.M.-V.); joseantonio.guerrero@inecol.mx (J.A.G.-A.); 5 Departamento de Ciencias Químico-Biológicas, Campus Hermosillo, Universidad de Sonora, Blvd. Luis Donaldo Colosio y Rosales s/n, Centro, Hermosillo 83000, Sonora, Mexico; robles.zepeda@unison.mx

**Keywords:** bioactive, agro-wastes, ganoderic acid, liquid culture, *Ganoderma*

## Abstract

**Background/Objective:** *Ganoderma* spp. have long been studied for their bioactive pharmacological properties, and their biomass and extracts have been obtained from various sources. This study adopts a novel approach: enriching a liquid culture of *Ganoderma mexicanum* with a vineyard pruning waste extract to identify bioactive compounds with antiproliferative activity through enriched chromatographic fractions. **Methods:** The ethanolic extract from a mycelial culture was separated following a partitioning process, and the hexane fraction was subsequently separated in a chromatographic column. The fractions were evaluated for their antiproliferative properties against cancer cell lines. The interactions of the molecules identified with peroxisome proliferator-activated receptor gamma (PPAR-γ) were analyzed via molecular docking. **Results:** Three chromatographic fractions (FH11–FH13) exhibited antiproliferative activity which was significantly more effective against non-small lung cancer cells (A549). The cells treated with the crude extract and fractions presented a balloon-like morphology. A chemical analysis of the active fractions allowed us to identify four compounds: one fatty acid (9-Hydroxy-10E,12Z-octadecadienoic acid) and three triterpenes (ganoderic acids DM, TQ, and X). These compounds showed interactions with the PPAR-γ receptor through molecular docking. **Conclusions**: *Ganoderma mexicanum* is a promising source of compounds with antiproliferative activity that could serve as natural ligands for PPAR-γ and has possible applications in lung cancer therapy.

## 1. Introduction

*Ganoderma* is a promising fungus for the treatment of multiple health conditions. This genus has great species diversity and, thus, their extracts have been analyzed for use against pharmacological issues [1,2]—with these extracts coming from different sources, such as basidiocarps, spores, sporoderm-broken spores, and mycelia [3,4,5]—due to their possible antiproliferative [6], cytotoxic [7], anti-inflammatory [8], hepatoprotective [8], sedative–hypnotic [9], antioxidant [10], antituberculosis [11], and anti-staphylococcus [12] effects.

Many studies have focused on identifying and isolating bioactive metabolites and searching for their potential therapeutic effects against various diseases, including cancer. The different mechanisms could respond differently to natural or synthetic ligands in cancer [13]. For instance, the water extract of sporoderm-broken spores of *G. lucidum* has been shown to inhibit the signal transducer and activator of transcription 3 (STAT3) that mediates STAT3-induced ferroptosis [5]. Gurbilek et al. [13] demonstrated that thymoquinone increases the levels of peroxisome proliferator-activated receptor gamma (PPAR-γ) protein, which can reduce cell viability and regulate the expression of tumor suppressor genes. The activation of PPAR-γ via its ligands could affect tumor progression via cellular processes [14]. PPAR-γ has one natural ligand (15-deoxy-D12,14-prostaglandin J2) but many synthetic ligands such as ciglitazone, troglitazone, pioglitazone, telmisartan, rosiglitazone, and others [15]. Advancing the discovery of novel natural ligands is crucial for developing more effective cancer therapies.

Exploring new natural ligands may offer promising alternatives for pharmacological applications, and identifying the bioactive metabolites of *Ganoderma* extracts is imperative from different perspectives [16,17,18]. Polysaccharides and triterpenoids are the metabolites in *Ganoderma* extracts that have been most studied. However, other less-studied chemical compounds, such as amino acids, fatty acids, sterols, proteins, organic acids, and steroids, are also constituents of their extracts [3,4]. Chafouz et al. [10] identified a fatty acid, three sterols, and two lanostane-type triterpenoids from the fruiting body of *G. adspersum*. Despite this species being one of the most studied, Zhang et al. [19] isolated ten undescribed lanostane triterpenoids from *G*. *lucidum*. Zhang et al. [20] reported that fraction C from the 50% ethanol extract of *G. neojaponicum* presents eight triterpenoids and has anti-inflammatory properties, inhibiting the oxidative activity of the NF-kB pathway. Chinthanom et al. [21] isolated eight lanostane triterpenoids from mycelial cultures of *G. weberianum*, three of which present anti-malaria properties.

Recent studies have focused on utilizing agro-wastes in solid and liquid basidiomycete cultures [6,22,23]. The Sonora state in northwestern Mexico is the primary source of grape production, yielding 307,153 tons, which accounts for 80.6 percent of the national total [24]. In a previous study, vineyard pruning waste (VPW) extracts enhanced the biomass production of native *Ganoderma* spp. strains from the Sonoran Desert [25]. Different sources, techniques, and strategies in liquid culture increase biomass and the synthesis of bioactive compounds [25,26]. Elicitors and culture conditions could modify and enhance the synthesis of compounds with a pharmacological approach such as methyl jasmonate (MeJa) in *G. applanatum* [27] and VPW extracts in *G*. *tuberculosum* [28], among others. The ethanolic extract from the fruiting bodies of *G*. *mexicanum* (previously *G*. *weberianum*) showed potential as an anti-inflammatory and antiproliferative agent against the A549 cancer cell line [29].

The study of bioactive *Ganoderma* compounds is no longer limited to *G. lucidum*. For the first time, the present work evaluates a liquid culture of *Ganoderma mexicanum*—a native strain from the Sonoran Desert—enriched with a vineyard pruning waste extract to promote biomass production and bioactive compounds with antiproliferative activity through enriched chromatographic fractions. In addition, whether these compounds can act as natural ligands for PPAR-γ was analyzed.

## 2. Results

### 2.1. Antiproliferative Activity of Ethanolic Extract

**Figure 1:**
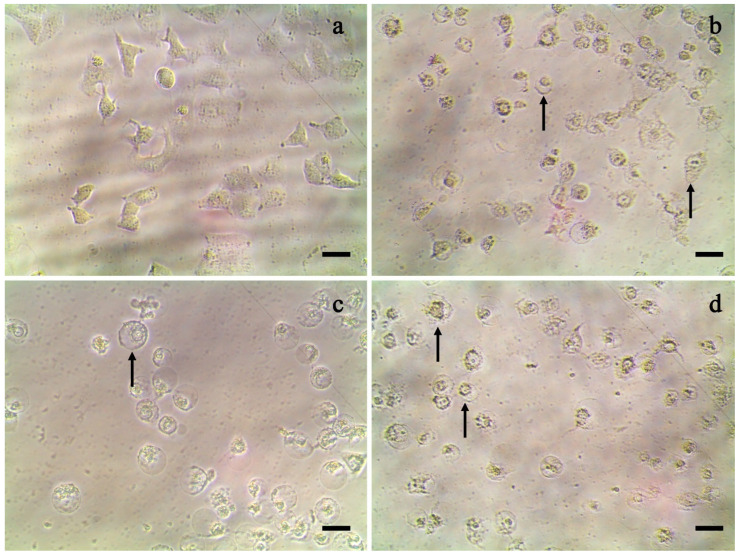
Morphological changes in the A549 cell line against *Ganoderma mexicanum* extracts. (**a**) Control, dimethyl sulfoxide (DMSO); (**b**) crude extract, 200 μg/mL; (**c**) hexane fraction, 200 μg/mL; and (**d**) ethyl acetate fraction, 200 μg/mL. Black arrow: morphological changes. Scale bar: 20 μM.

The ethanolic crude extract, hexane fraction (FH), and ethyl acetate fraction (FEA) showed high antiproliferative activity at 200 μg/mL against the A549 cancer cell line (Figure 1), where all extracts and fractions produced the same morphological changes, resembling balloon-like cells, in the A549 cell line. The crude extract showed no significant difference in the 12.5–100 μg/mL range (Figure 1b). FH presented an effect at 200 μg/mL and a decrease in cell viability at 25 and 100 μg/mL (Figure 1c). FEA demonstrated a better impact at 100 μg/mL on the A549 cell line (Figure 1d). The best yield during liquid–liquid separation occurred with FH. Neither FH nor FEA affected the HeLa cancer and ARPE-19 non-cancerous cell line. These achievements show that the crude extracts and fractions could have a specific effect against the A549 cell line with an IC_50_ between 107.84 and 144.92 μg/mL (Table 1).

### 2.2. Antiproliferative Activity of Hexane Fraction

FH leads to 13 fractions (FH1–FH13), but only FH11–FH13 had antiproliferative effects against the A549, HeLa, MDA-MB-231, and ARPE-19 cell lines (Table 2). The fractions were more effective against the A549 cell line, with an IC_50_ between 78.4 and 96.1 μg/mL. In the HeLa cells, the IC_50_ for the different fractions was between 106.5 and 148.7 μg/mL; for MDA-MB-231, it was 162 to 200 μg/mL. The fractions were less effective against the non-cancerous ARPE-19 cell line (>200 μg/mL), which could be associated with their selectivity for cancer cells (Table 2).

### 2.3. Characterization of Hexane Fraction

**Figure 2:**
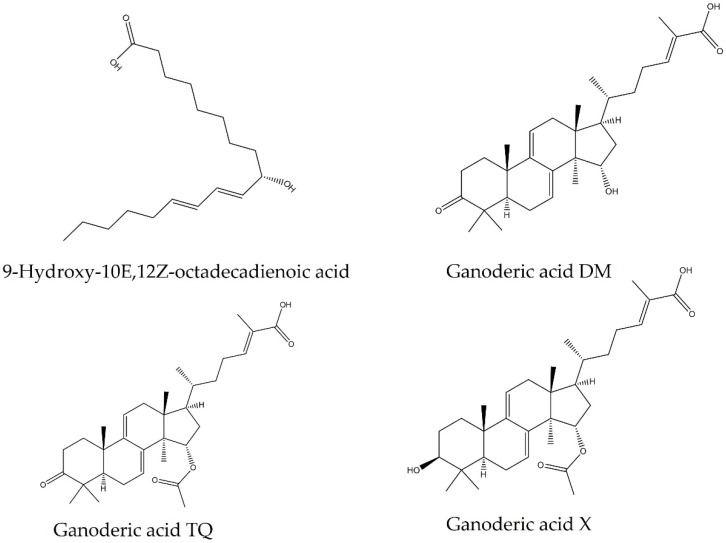
Molecular structure of fatty and ganoderic acids (GA-DM, GA-TQ, and GA-X).

**Figure 3:**
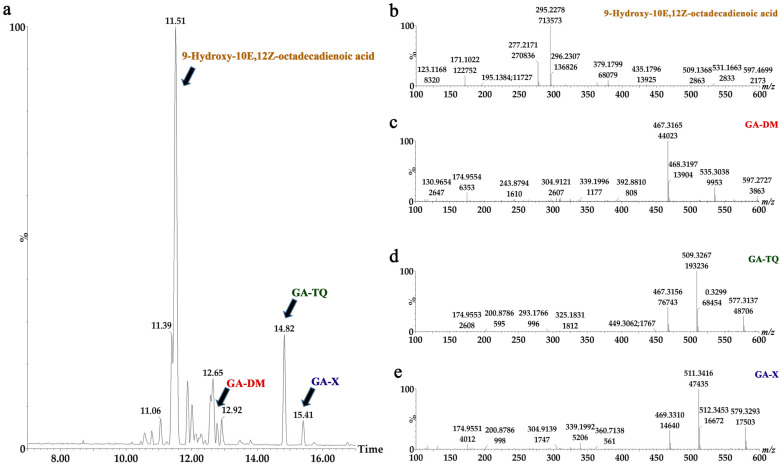
LC-MS analysis from fraction 12 of *Ganoderma mexicanum*. (**a**) UHPLC-ESI-TOF-MS of FH12. Mass spectra of (**b**) 9-Hydroxy-10E,12Z-octadecadienoic acid, (**c**) GA-DM, (**d**) GA-TQ, and (**e**) GA-X.

**Figure 4:**
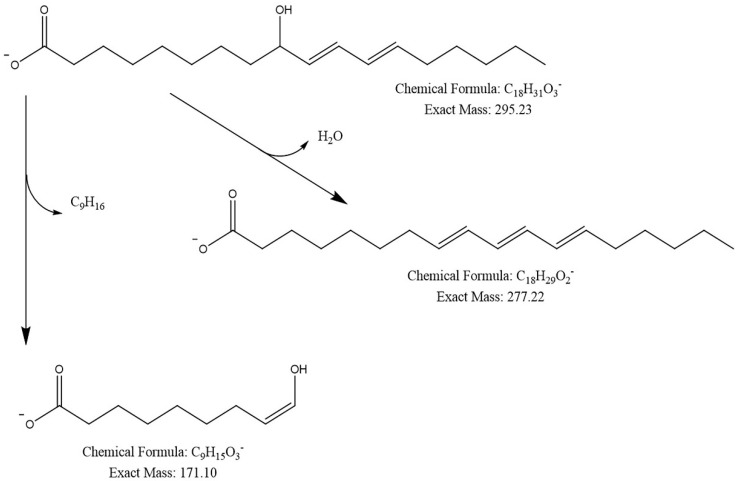
Fragmentation pattern of 9-Hydroxy-10E, 12Z-octadecadienoic acid.

Four compounds were identified: three triterpenoids and one fatty acid (Figure 2). Hexane fractions 11 and 12 have three similar compounds: 9-Hydroxy-10E,12Z-octadecadienoic acid, and two ganoderic acids (GA-X and GA-TQ; Table 3). FH12 was selected due to its high yield in consecutive experiments. Figure 3a shows a chromatogram of fraction 12, with the peaks of the four identified compounds and their mass spectra: Figure 3b, 9-Hydroxy-10E,12Z-octadecadienoic acid; Figure 3c, GA-DM; Figure 3d, GA-TQ; and Figure 3e, GA-X. Figure 3b shows the fragmentation pattern of the most abundant compound of FH12. The molecular ion was *m*/*z* 295.2278 [M-H]^−^, corresponding to the molecular formula C_18_H_31_O_3_, with a mass error of 0.61 ppm. Likewise, the fragmentation pattern with fragment *m*/*z* 277.2171 [M-H_2_O-H]^−^ and molecular ion *m*/*z* 171.1022 [M-C_9_H_16_-H] corresponds to 9-Hydroxy-10E,12Z-octadecadienoic acid (Figure 3b and Figure 4).

### 2.4. Molecular Docking

**Figure 5:**
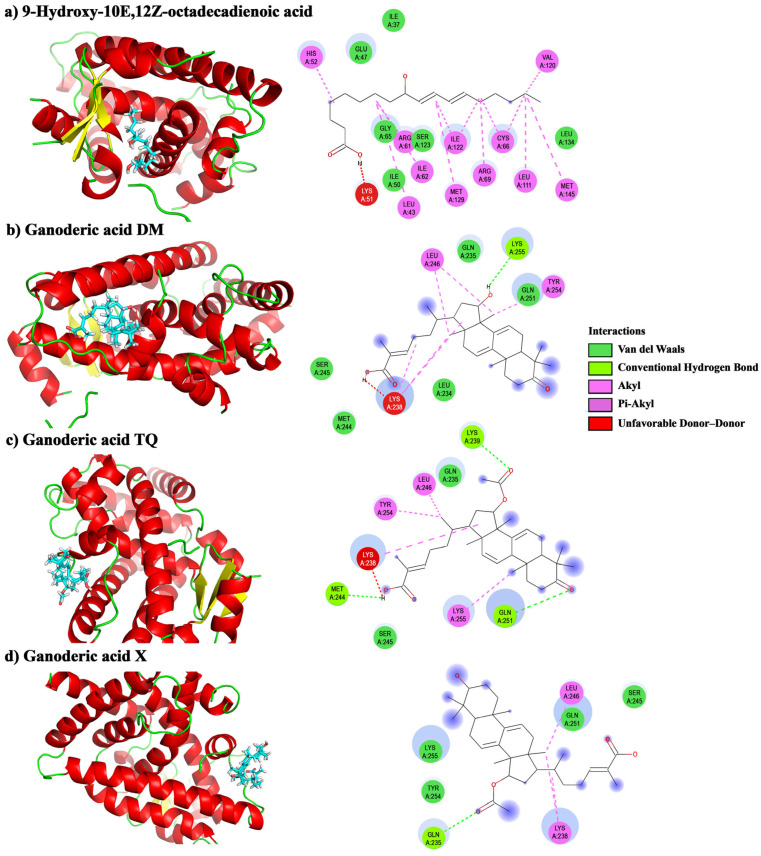
Molecular interactions between *Ganoderma* molecules and chain A of PPAR-γ.

The molecular interactions of PPAR-γ with the 9-Hydroxy-10E, 12Z-octadecadienoic acid and the three ganoderic acids were analyzed. The calculated binding affinity was −5.74 kcal/mol for 9-Hydroxy-10E, 12Z-octadecadienoic acid, −6.6 kcal/mol for GA-X, −6.8 kcal/mol for GA-DM, and −6.7 kcal/mol for GA-TQ. 9-Hydroxy-10E, 12Z-octadecadienoic acid exhibited alkyl and π-alkyl interactions with HIS^52^, LEU^43^, ARG^61^, ILE^62^, CYS^66^, ARG^69^, LEU^111^, VAL^120^, ILE^122^, MET^129^, and MET^145^ from chain A of PPAR-γ (Figure 5a). The interactions between GA-DM and PPAR-γ revealed a hydrogen bond between LYS^255^ and the hydroxyl group at C15. Van der Waals, alkyl, and π-alkyl interactions were present in GA-DM (Figure 5b). Ganoderic acid TQ formed three hydrogen bonds between GLN ^251^ and the Keto group at C3, LYS ^239^ and the acetoxy at C15, and MET^244^ and the carboxyl group at C32. It also exhibited Van der Waals, alkyl, and π-alkyl interactions (Figure 5c). GA-X also showed Van der Waals interactions with LYS^255^, TYR^254^, GLN^251^, and SER^245^, as well as alkyl interactions between LEU^246^ and LYS^238^. A hydrogen bond was observed between the GLN^235^ from chain A and the acetyloxy group at C15 of GA-X (Figure 5d). These positions have low energy despite unfavorable donor–donor interactions.

## 3. Discussion

**Figure 6:**
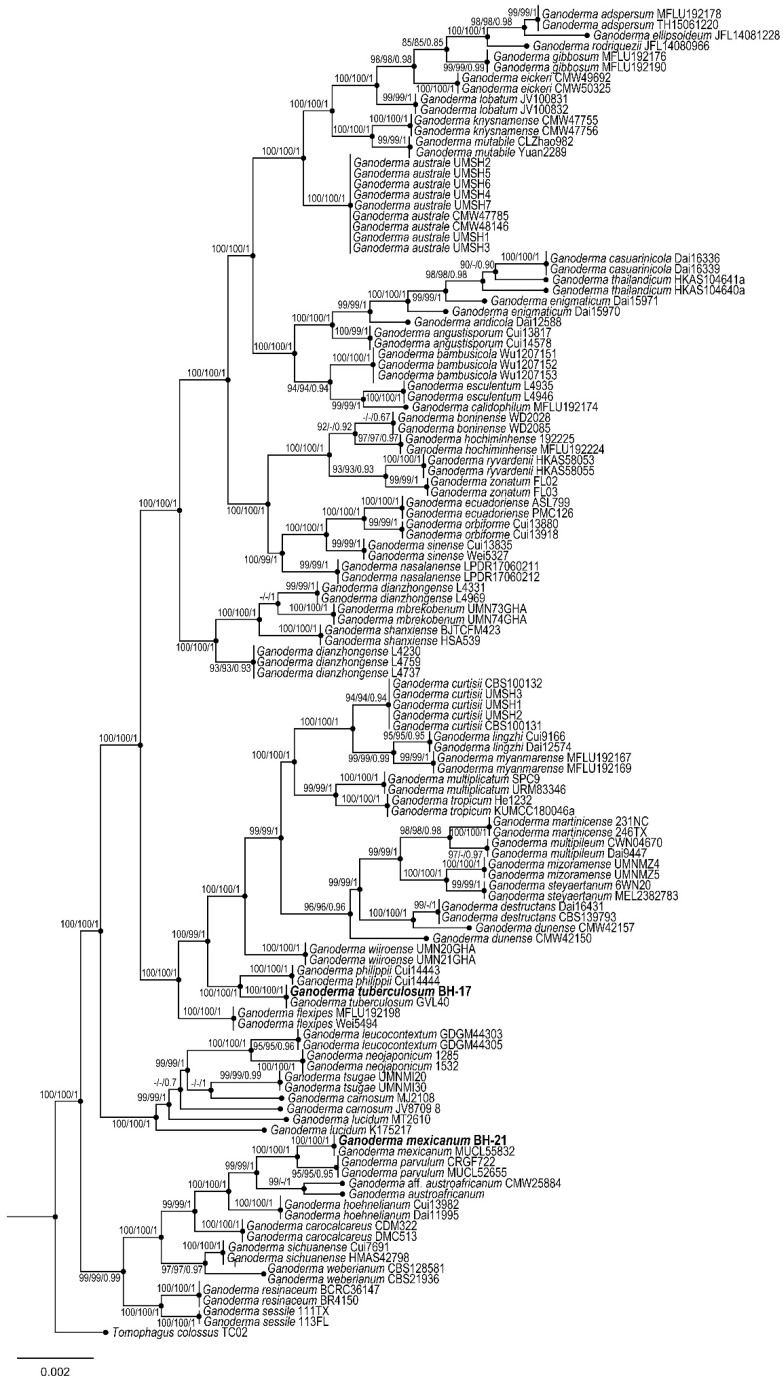
Phylogenetic tree based on a concatenated ITS, *rpb2*, and *tef1*-α dataset from a *Ganoderma* strain (BH-21).

The native strain of *Ganoderma mexicanum* from the Sonoran Desert (Figure 6) was selected for evaluation due to its biomass enhancement and metabolic response when grown in a liquid culture with a vineyard pruning waste extract [25,30]. This species grows in Argentina, Brazil, Martinique, and Mexico [31]. Most *Ganoderma* extracts use ethanol (90–99%), but the extraction methodology varies [18,32]. In this study, sonication was performed for 30 min and incubation for 24 h to improve the extraction yield. The *G. mexicanum* ethanolic extracts showed a dose-dependent effect on the cell viability of the A549 cancer cell line, which is characteristic of *Ganoderma* extracts [4].

A balloon-like morphology was observed (Figure 1), which was also reported by Zhong et al. [33]. They mentioned that these swelling features are characteristic of pyroptosis, a type of cell death in breast cancer cells. Pyroptosis occurred in response to the *G. lucidum* extract treatment used at 20 to 200 μg/mL, where the therapy increased the abundance of reactive oxygen species, activated caspase-3, and augmented the number of pores in the cell membrane due to the gasdermin E (GSDME) protein. Caspase-3 can cleave and activate GSDME, converting apoptosis to pyroptosis in cells that highly express GSDME [34]. Pyroptosis has been redefined as a gasdermin-mediated programmed necrotic cell death [35]. A photocatalytic superoxide radical generation strategy enables the activation of light-controlled pyroptosis in cancer cells via the caspase-3/GSDME pathway, potentially facilitating the application of opto-controlled pyroptosis in cancer therapy [36]. Pyroptosis-targeted therapy shares similarities with chemotherapy but with reduced side effects, being a potential approach for anti-tumor immunotherapy [37]. Likewise, the activation of pyroptosis can rescue chemotherapy-resistant lung and pancreatic cancer cells, thereby overcoming chemotherapy resistance in cancer [38]. Further research is needed to understand how cancer-associated pyroptosis, mediated by GSDMB, C, and E, is regulated [35]. The *G. mexicanum* ethanolic extracts tested in this study on the A549, HeLa, and MDA-MB-231 cell lines could cause pyroptotic cell death. Still, confirming this type of cell death with other studies is necessary, as ethanol extracts could also affect healthy cell lines. Similarly to the study of *G. lucidum*, the ethanolic extract of mycelia showed cytotoxicity against Vero cells [18]. Therefore, this study’s *G. mexicanum* ethanolic extract is selective to cancer cell lines as it did not affect the non-cancerous ARPE-19 cell line.

Other studies reported antiproliferative activity from *Ganoderma* spp. extracts or isolated compounds against the A549 cell line. Chloroformic extracts from the fruiting bodies of *G. subincrustatum* and *G*. *weberianum* exhibited IC_50_ of <25 and 42.8 μg/mL against the A549 cells, respectively [6]. Treatment with 40 µM of GA-DM resulted in cell viabilities of 66, 47, and 1.8% at 24, 48, and 72 h, respectively, in the A549 cancer cell line [39]. Nine triterpenoids from the fruiting bodies of *G*. *lucidum* demonstrated an IC_50_ of 24.63 μg/mL [40]. These findings reflect a common pattern of dose- and time-dependent effects of the *Ganoderma* extracts and are consistent with the results of the present study.

Three triterpenoids and one fatty acid were identified in FH, where the abundant peaks in the fractions correspond to 9-Hydroxy-10E, 12Z-octadecadienoic acid. The cytotoxic effect of this fatty acid against the mouse p388 cell line has been reported; this compound was isolated for the first time from rice bran [41]. Fatty acids are essential in the metabolism of fungi due to their importance as a metabolic energy source, building phospholipids, and reservoirs. They are involved in growth due to their continuous modifications to the membrane [42]. *Ganoderma* oils have recently been studied and shown to have multiple applications. A study showed that *G. lucidum* spore oil (ganooil) could improve immune activity in mice. Additionally, using ganooil with cyclophosphamide helps to suppress lung metastasis in the case of circulating breast cancer cells [43]. *G. lucidum* spore oils decrease inflammation, accelerate skin wound healing in mice, and regulate the microbiota [44]. This helps to increase the possibility of using *Ganoderma* extracts in a pharmacological approach.

Many research studies have looked at the pharmacological application of ganoderic acids, some of which have analyzed the activity on cancer cells of the compounds identified in this study. The patent JP-2019163292-A is an extract of *G. lingzhi* that presents anti-influenza activity through neuraminidase inhibition [45]. This extract comprises 33 ganoderic acids, including GA-TQ and GA-DM [41]. GA-DM promotes autophagic flux and cytotoxic effects against non-small-cell lung cancer by inhibiting the Akt/mTOR pathway [39]. This study could be applied to the *G. mexicanum* ethanolic extract because some of these ganoderic acids are present in its extracts and fractions. Many studies, including in vivo assays, gene expression analyses, and flow cytometric evaluations, are required to validate the pharmacological application of these extracts.

In 2022, lung cancer ranked first in global incidence, accounting for 18.7% of cancer-related mortality, with 1.8 million deaths worldwide. Its incidence in both males (15.3%) and females (9.4%) is higher than that of other types of cancer [46]. PPAR-γ, a member of the steroid receptor superfamily, has recently been implicated in lung cancer. PPAR-γ is a ligand-activated transcription factor involved in various processes, including cell differentiation, lipid and glucose metabolism, energy homeostasis, and inflammation [14,15]. A key feature of PPAR-γ is its binding region. Shang et al. [47] investigated the activation and repression of this binding site, where the orthosteric ligands compete with the repressive helix 12 conformations for binding to the ligand-binding pocket. In this study, the fatty acids were bound near the pocket; conversely, the ganoderic acids were positioned in the allosteric form. Gill et al. [48] mentioned that the keto and hydroxyl groups play roles in signaling through different receptors. GA-DM contains a hydroxyl group at C15, while GA-TQ possesses a keto group at C3; these functional groups could be related to the ability of these molecules to interact with PPAA-γ [48].

Notably, PPAR-γ mRNA is expressed in most lung cancer cell lines, including the A549 cell line [49]. Various PPAR-γ ligands have been shown to induce cell differentiation and apoptosis and to inhibit cell growth in lung cancer cells [14]. Arachidonic acid-induced PPAR-γ activation suppressed the A549 cell growth and NF-κB binding activity, whereas it decreased aldehyde dehydrogenase 3A1 expression and increased lipid peroxidation [50]. PPAR-γ/NF-κB axis (upstream) and the IL-6/IL-6R (downstream) signaling pathway of aquaporin 3-mediated M2 macrophage polarization could regulate the proliferation, migration, and glycometabolism of lung adenocarcinoma [51]. PPARγ inhibits the tumor immune escape of non-small-cell lung cancer (NSCLC) by inducing programmed death ligand 1 (PD-L1) protein autophagic degradation in lysosomes, suppressing NSCLC tumor growth by increasing T-cell activity [52]. The anti-inflammatory agent (ACT001) significantly ameliorated inflammation and pyroptosis via the PPAR-γ/NF-κB signaling pathways in lipopolysaccharide-induced NR8383 alveolar macrophages [53]. A strong anti-inflammatory effect of the natural compound honokiol was associated with the PPAR-γ–TLR4–NF-κB activation signaling pathway and gasdermin-D-mediated macrophage pyroptosis [54].

Likewise, PPAR antagonists could provide novel therapeutic options, combined with conventional anticancer drugs and the emerging immunotherapy [55]. However, the use of synthetic PPAR agonists, such as rosiglitazone, has been associated with cardiovascular toxicity, due to the downregulation of cyclooxygenase-2 (COX-2) in response to PPAR-γ ligand signaling in lung cancer [56]. Although PPARγ activation in myeloid cells promotes lung cancer progression and metastasis [57], it remains a viable target for lung cancer treatment and prevention as monotherapy and in combination with traditional radiotherapy or chemotherapy [15]. Therefore, identifying natural ligands with antiproliferative activity is crucial for developing safer therapeutic options. Fatty and ganoderic acids identified in *G. mexicanum* may be promising natural PPAR-γ ligands for targeting lung cancer cell lines. Moreover, these extracts and fractions do not exhibit cytotoxic effects on non-cancerous ARPE-19 cells. Further studies are therefore necessary to validate their possible pharmacological applications and to elucidate their potential role in PPAR-γ signaling.

## 4. Materials and Methods

### 4.1. Fungal Culture

The strain *Ganoderma mexicanum* (BH-21) was acquired from the Plant and Fungi Biotechnology Laboratory of the Research Center in Food and Development A.C. The species was identified by studying the morphological characteristics of the fruiting body and phylogenetic inferences based on a concatenated ITS, *rpb2*, and *tef1-α* dataset of genomic DNA extracted from the strain’s mycelial culture in a Petri dish with PDA. The methods of Cabarroi-Hernández et al. [31] were followed with some modifications. The strain was cultured in a cornmeal medium with modifications, including the vineyard pruning waste extract (VPW), 2.9 g of peptone, 16 g of glucose, 21 g of cornmeal flour, and 7 g of soy protein per 1 L of distilled water [58]. The cornmeal flour was boiled for 15 min, filtered, and cooled before adding the other components; the initial pH was 5.5. The medium (400 mL) was cultured in a 1 L flask; each inoculum contained eight agar disks. The culture strategy led to growth for five days until the addition of 1500 μg/L of polar and non-polar VPW extracts (aqueous–ethanol/toluene–chloroform, 3:1) [25]. The flask was cultured at 25 °C under agitation in darkness on a refrigerated orbital incubator shaker (TE-421, Tecnal, São Paulo, Brazil) and collected after up to five days per treatment. The liquid culture was eliminated via filtering with Whatman filter paper #1 and washed with distilled water. The biomass was stored at −20 °C for lyophilization (LyoQuest-85 PLUS ECO, Lyoquest, Telstar, Terrassa, Spain). The biomass was weighed and stored at 25 °C.

### 4.2. Extraction and Isolation

The biomass of *G. mexicanum* was used to obtain the ethanolic extract. This extract was prepared in a 1:10 (*m*/*v*) ratio, using 100 g of mycelial biomass and 1 L of ethanol ABS ACS. The biomass was homogenized and sonicated for 1 h (Ultra Sonic Cleaner, Branson 2210R-MT, Branson Ultrasonics, Brookfield, CT, USA), with a power of 210.6 W and an operating frequency of 40 kHz. The extract was left overnight at room temperature and filtered with Whatman filter paper #1. This procedure was performed in triplicate. Then, the ethanolic extract was dried under reduced pressure (Rotary evaporator BÜCHI R-200, Flawil, Switzerland) and placed in crystal dishes at 45 °C in the oven. Later, the extract was weighed to determine the yield.

The ethanolic extract of mycelial *G. mexicanum* was fractioned with n-hexane and ethyl acetate via partitioning (liquid–liquid extraction). The extract (9 g) was suspended with H_2_O and sequentially partitioned with n-hexane and ethyl acetate (CTR Scientific, Monterrey, Mexico) to generate a hexane fraction (FH, 3.5 g), an ethyl acetate fraction (FEA, 0.9 g), and a residual fraction (FR, 4.6 g). An aliquot of FH was sub-fractionated with a chromatography column (5.5 × 50 cm) using Silica Gel 60 (200-400 mesh, Sigma-Aldrich, Saint Louis, MO, USA). The mobile phase was ethyl acetate–methanol in different proportions *v*/*v*: 100:0, 95:5, 75:25, and 0:100. Then, 10 mL fractions were collected and analyzed via thin layer chromatography (TLC). Ultraviolet light (254/366 nm) was used to visualize the spots, and the spots were stained with *p*-anisaldehyde–H_2_SO_4_–EtOH (1:1:98), followed by heating at 110 °C until positive areas were revealed.

### 4.3. UHPLC-ESI-TOF-MS

The fraction samples were analyzed in a UPLC Class I of Waters coupled to a Synapt G2Si HDMi mass spectrometer (Waters, Milford, MA, USA). The analysis was performed in an Acquity BEH column (1.7 μm, 2.1 × 50 mm); the temperature of the column was 40 °C, and that for the samples was 15 °C. The mobile phases were water (A) and acetonitrile (B), both with 0.1% formic acid (Sigma-Aldrich, USA). The gradient conditions of the mobile phases were a 20 min linear gradient with 20–99% B, 4 min with 99% B isocratic, and a 1 min linear gradient with 90–20% B (total run time: 30 min). The flow rate was 0.3 mL/min; the sample injection contained 5 μL. The mass spectrometric analysis was performed with an electrospray ionization source in negative mode with a capillary (2500 V), sampling cone (40 V), and source offset (80 V); the source temperature was 120 °C, and the desolvation temperature was 20 °C. The desolvation gas flow was 600 L/h, and the nebulizer pressure was 6.5 Bar. Leucine-enkephalin was used as the lock mass (554.2615, [M-H]^−^), and Ganoderic acid A (Sigma-Aldrich, USA) and oleanolic acid (Sigma-Aldrich, USA) were used as the standards. The conditions used for the MS^E^ analysis were a mass range of 50–1200 Da, function 1 CE 6 V, function 2 CER 10-30 V, and a scan time of 0.5 s. The data were processed with MassLynx (version 4.1) software [59]. The mass search was conducted using the food database (https://foodb.ca/spectra/ms/search, accessed on 12 June 2023).

### 4.4. Cell Lines and Culture Conditions

The human lung adenocarcinoma (A549), human cervical adenocarcinoma (HeLa), human breast cancer (MDA-MB-231), and non-cancerous retinal pigment epithelium (ARPE-19) cell lines were purchased from American Type Culture Collection (ATCC, Manassas, VA, USA). The cell lines were cultured in Dulbecco’s Modified Eagle Medium (Sigma Aldrich, USA) supplemented with 5% heat-inactivated fetal bovine serum and penicillin (100 U/mL) in 25 cm^2^ culture dishes. The cells were grown in an incubator at 37 °C in a 5% CO_2_ atmosphere under sterile conditions with 95% relative humidity [60].

### 4.5. Antiproliferative Activity

An MTT (3-(4,5-dimethylthiazol-2-yl)-2,5-diphenyltetrazolium bromide, Sigma-Aldrich, USA) assay was used to evaluate the antiproliferative activity of the Ganoderma extracts and fractions. The dried extract samples were diluted in DMSO at 40 mg/mL. A serial dilution (200 to 25 µg/mL) was performed from this solution. The cell suspension (200,000 cells/mL) was placed on a 96-well plate (Costar, Corning, NY, USA). The cells were incubated for 24 h; then, 50 µL of the extract or fraction was placed in each well and incubated for another 48 h. The wells were washed with PBS 1×, 100 µL of the culture medium prepared with MTT (9:1) was added and incubated for 4 h, and 100 µL of acidified isopropyl alcohol was used to dissolve the formazan crystals. After 10 min in darkness, the absorbance was read with a dual-wavelength plate spectrophotometer at 630 and 570 nm (iMark microplate absorbance reader, Bio-Rad, Lab., Mexico City, Mexico). The percentage of cellular viability was calculated using the Excel software package from Microsoft Office 365 [61].

### 4.6. In Silico PPAR-γ Interaction

The three-dimensional structure of the target protein (6ONJ, crystal structure of the PPAR-γ ligand-binding domain in complex with the TRAP220 peptide and agonist rosiglitazone) was obtained from the Protein Data Bank (PDB, https://www.rcsb.org/, accessed on 8 March 2025) [47]. The structure was preprocessed by removing unnecessary water molecules and nonessential ligands and refined using structural tools. Hydrogen was added, and the appropriate protonation states were adjusted to physiological pH using UCSF Chimera 1.17.1. The structures of the various ligands to be tested were created in ChemDraw Professional 15.0.0.106. Each ligand structure was optimized with Avogadro V1.2.0. software, where the protonation state of the molecules was modified to simulate physiological pH. AutoDock Vina: 1.2.7 software was utilized for blind docking. A grid box was defined to encompass the entire surface of the protein, ensuring the thorough exploration of the potential binding sites. The configuration parameters included a grid spacing of 1.0 Å and a box size sufficient to cover the entire region of the macromolecule. Binding free energy (∆G) values were measured to identify the lowest-energy conformations likely to form interactions, which were subsequently analyzed using PyMOL Molecular Graphics System, Version 3.1 and UCSF Chimera. The Protein–Ligand Interaction Profiler (PLIP) tool was employed to identify hydrogen bonds, hydrophobic interactions, and salt bridges [62,63,64].

## 5. Conclusions

A native strain of *Ganoderma mexicanum* from the Sonoran Desert cultivated in liquid culture with an extract of vineyard pruning waste was proven to be an effective source of bioactive compounds (e.g., 9-Hydroxy-10E,12Z-octadecadienoic acid and the ganoderic acids GA-DM, GA-TQ, and GA-X), which could have a synergic effect against cancer cell lines. Moreover, it was demonstrated that the crude ethanolic extract and the hexane and ethyl acetate fractions do not affect non-cancerous cell lines. Molecular docking showed that the four identified molecules interact with PPAR-γ, which is implicated in lung cancer. These compounds could serve as a promising source of natural ligands for PPAR-γ and have possible applications in lung cancer therapy.

## Figures and Tables

**Table 1:** Antiproliferative activity of *Ganoderma mexicanum* under the effect of the ethanolic crude extract and fractions from the liquid–liquid extraction. Cell lines’ IC_50_ (μg/mL) represents the average of three independent experiments with standard deviation (±SD).

Treatments	A549	HeLa	ARPE-19
Ethanolic crude extract	144.96 ± 9.4	>200	>200
Hexane fraction	140.84 ± 17.9	>200	>200
Ethyl acetate fraction	107.842 ± 9.4	>200	>200
Residual fraction	>200	>200	>200

**Table 2:** Antiproliferative activity of *Ganoderma mexicanum* hexane fraction. Cell lines’ IC_50_ (μg/mL) represents the average of three independent experiments with standard deviation (±SD). FH: hexane fraction.

Fraction	A549	HeLa	MDA-MB-231	ARPE-19
FH1–FH10	>200	>200	>200	>200
FH11	78.4 ± 7.1	106.5 ± 2.9	162 ± 9.8	>200
FH12	96.1 ±12.8	148.8 ± 8.5	>200	>200
FH13	89.3 ± 4.2	136.6 ± 10.4	181.5 ± 9.6	>200

**Table 3:** Compounds identified in the hexane fraction of *Ganoderma mexicanum* mycelial ethanolic extract. FH: hexane fraction.

Fraction	Molecules	Formula	Mass	*m*/*z*	RT
FH11	9-Hydroxy-10E,12Z-octadecadienoic acid	C_18_H_31_O_3_	296.64	295.22	11.39
	Ganoderic acid TQ	C_32_H_46_O_5_	510.70	503.32	14.82
	Ganoderic acid X	C_32_H_48_O_5_	512.35	511.34	15.40
FH12	9-Hydroxy-10E,12Z-octadecadienoic acid	C_18_H_31_O_3_	296.64	295.22	11.52
	Ganoderic acid DM	C_30_H_44_O_4_	468.70	467.31	12.78
	Ganoderic acid TQ	C_32_H_46_O_5_	510.70	509.32	14.82
	Ganoderic acid X	C_32_H_48_O_5_	512.35	511.34	15.39
FH13	9-Hydroxy-10E,12Z-octadecadienoic acid	C_18_H_31_O_3_	296.64	295.22	11.51
	Ganoderic acid DM	C_30_H_44_O_4_	468.70	467.31	12.77
	Ganoderic acid X	C_32_H_48_O_5_	512.35	511.34	15.41

## Data Availability

Data is contained in the paper.

## Abbreviations

The following abbreviations are used in this manuscript:

STAT3	signal transducer and activator of transcription 3
PPAR-γ	peroxisome proliferator-activated receptor gamma
VPW	vineyard pruning waste
FH	hexane fraction
FEA	ethyl acetate fraction
GA	ganoderic acid
GSDME	gasdermin E
Caspase	cysteine aspartate specific proteases
Ganooil	*Ganoderma lucidum* spore oil
NF-κB	nuclear factor—kappa B
IL-6/IL-6R	interleukina-6/interleukina-6 receptor
NSCLC	non-small-cell lung cancer
PD-L1	programmed death ligand 1
TLR4	Toll-like receptor 4
COX-2	cyclooxygenase-2
DMSO	dimethyl sulfoxide
MTT	3-(4,5-dimethylthiazol-2-yl)-2,5-diphenyltetrazolium bromide
MeJa	methyl jasmonate
